# Effects of Atorvastatin on Negative Sign in Chronic Schizophrenia: a Double Blind Clinical Trial

**Published:** 2015

**Authors:** Mehdi Sayyah, Hatam Boostani, Mitra Ashrafpoori, Siroos Pakseresht

**Affiliations:** a *Faculty member of Education Development Center (EDC), Psychiatrist, Ahvaz Jundishapur University of Medical Sciences, Ahvaz, Iran. *; b *Faculty member of Psychiatry Group, Psychiatrist, Ahvaz Jundishapur University of Medical Sciences, Ahvaz, Iran. *; c *Resident of psychiatry, Ahvaz Jundishapur University of Medical Sciences, Ahvaz, Iran.*

**Keywords:** Schizophrenia, Negative sign, Atorvastatin, Clinical trial

## Abstract

The aim of this study was to evaluate the effects of Atorvastatin on negative symptoms in patients with chronic schizophrenia. The study was a prospective, double-blind, 6-week trial. Forty patients participated in the study; 19 patients were assigned to the Atorvastatin group as well as 21 patients to the placebo group. For assessing negative signs, we used Scale for the Assessment of Negative Symptoms (SANS) in weeks 1st, 3nd, 4th, and 6th. Moreover, patients were randomly assigned to treatment groups with Risperidone (6 mg/day) plus 20 mg Atorvastatin or with Risperidone (6 mg/day) plus placebo. Mean scores of Scale for the Assessment of Negative Symptoms (SANS) decreased during the treatment but there was no significant difference between the mean scores of two groups. The result of this trial suggested that Atorvastatin can be effective in reducing negative sign in schizophrenia although further studies seem to be needed.

## Introduction

Schizophrenia is considered as a serious mental disorder with 1% prevalence rate (1) and with varied positive and negative signs and symptoms. Negative sign includes blunted affect, apathy, poverty of thought or speech, thought-blocking and social withdrawal (2). However conventional antipsychotic and atypical antipsychotic drugs improve signs and symptoms of schizophrenia, the treatments of negative sign generally show little benefit (3).

One of the hypotheses made to explain the pathophysiology of schizophrenia is inflammation in the brain. Evidences show that inflammation can be due to psychotic disorder such as schizophrenia (1, 4-5).

Another theory about development, progression, and treatment of schizophrenia is a Nitric Oxide role in the brain (2, 6). Some of evidence suggests that no system has influence in treatment and progression in schizophrenia (3, 7).Moreover, Atorvastatin is used in a group of drugs called 3-hydroxy-3-methylglutaryl coenzyme A (HMG CoA) reductase inhibitors, or statins (8) and Atorvastatin is used usually on treating high cholesterol. It also treats high triglycerides and prevents cardiovascular disease too.

The recent studies on Atorvastatin explain that it has potential mechanisms endothelial on nitric oxide syntheses (4, 9) and Suppression of inflammation (5, 10).

Considering the important roll of inflammation (1) and dysregulation in NO (2) through theories in the treatment and etiology of negative sign in schizophrenia and the effects of Atorvastatin on inflammation (5) and NO (4), therefore authors decided to exam Atorvastatin’ effect on the negative sign in schizophrenia in a double-blind clinical trial.

## Experimental

*Materials and Methods*

*Trial design*

The study was a six-week prospective, double-blind, and placebo-controlled trial. Two parallel groups of outpatients with chronic schizophrenia were randomly selected from Ahvaz Golestan Hospital and these population participated in the study from March, 2012 to December, 2012.

*Participants and setting*

Those participants were eligible for the study whether they met Diagnostic and Statistical Manual of Mental Disorders (DSM-IV-TR) criteria for Schizophrenia (11) and their scores were 50 or above in Positive and Negative Syndrome Scale (PANSS) (13) as well as 10 or above in Scale for the Assessment of Negative Symptoms (SANS) (13) too. For inclusion criteria, patient should be 25–65 years old and at least 5 years diagnosis schizophrenia. During the past three months before the beginning of the study, patients did not receive any long acting antipsychotic medication and lithium, and they were given written informed consent for participation in the study. Subjects were disqualified if they had suicidal thoughts and did not have any other psychiatric or neurological disorder and the patients were also assessed for significant cardiac, renal or hepatic diseases, history of sensitivity to medications with the station, pregnancy, lactation, and mental retardation. The presence of any of these problems caused patients not to meet the criteria for entering the study. Due to less side effects and the easy availability, Atorvastatin was selected among the existing statins.

The patients and their legally authorized representatives have provided written informed consent under the procedures defined by Jundishapur University of medical science. Moreover, the trial was performed in accordance with the Declaration of Helsinki and its latest revisions. The trial was registered in the Iranian registry of clinical trials (registration no. IRCT201202067290N1). In order to preserve the double-blind condition, Atorvastatin and placebo were dispensed in identical-appearing capsules. Atorvastatin capsules were filled with 20 mg and talcum powder while placebo capsules were only filled with talcum powder.

*Procedures*

After written informed consents were gathered from the patients and legally were authorized representatives, participants entered in a parallel group through randomized, double blind, fixed-schedule, six-week clinical trial. Through using a computer-generated list of random numbers, patients were randomly assigned to the treatment group with Risperidone (6 mg/day) with 20 mg Atorvastatin or Risperidone (6 mg/day) with placebo; Biperiden used maximum 4mg/day if patients have any extra pyramidal sign. The populations were 40 patients those enrolled in the study; 19 and 21 patients were assigned as the Atorvastatin and placebo groups, respectively (Table 1). It should be mentioned that no other psychotropic medication was prescribed. Moreover, participants did not receive any concomitant psychological treatment or psychosocial support. At the time of this study none of the patients received the psychiatric medication except Risperidone or Bipiridine and they were excluded in case of using a drug interfered with either Risperidone or Atorvastatin. The patients were examined by a psychiatry resident before the trial and during first, third, fourth, and sixth weeks. For assessing negative sign, we used Scale for the Assessment of Negative Symptoms (SANS). Treatment-induced adverse effects were assessed systematically per section of visiting by a score sheet that was designed for the present study and Liver function test (SGOT and SGPT) and creatinine tested in the fourth week.

**Table 1:** Demographic data of patients

**Characteristics**		**Placebo group (21)**	**Atorvastatin group (19)**	**P**
Sex	Female	11	9	1.00 (Chi-Square test)
	Male	10	10	
Marital status	Married	7	5	0.72 (Fisher Exact test)
	Single	14	15	
Age (Mean ± SD)		39.05 ± 10.31	34.75 ± 7.15	0.134 (2-independent t-test)
SANS (Week 0)		56.9 ± 7.4	53.1 ± 3.2	0.929 (2-independent t-test)

*Statistical analysis*

Data were collected and analyzed using intention-to-treat analyses. Repeated measures analysis of variance (ANOVA) was used to assess the effects of treatment (two study groups), time (weeks of the visit), and interaction of treatment and time. Significance of difference in mean scores in the first visit and duration of treatment was assessed by un-paired Student's t-test. The frequency of treatment-induced adverse effects in the study groups were analyzed by Fisher's exact test. All statistical tests were two-sided, and were considered statistically significant at P < 0.05.

## Results and Discussion

*Demographic characteristics*

Forty patients were enrolled in the study and divided in two groups; 19 members were assigned to the Atorvastatin group and other 21 to the placebo group (Table 1). The characteristics of two groups were summarized and showed in Table 1. The two groups were well matched and there were no statistically significant differences between the groups in demographic or a base score of SANS.

*Attrition*

**Figure 1:**
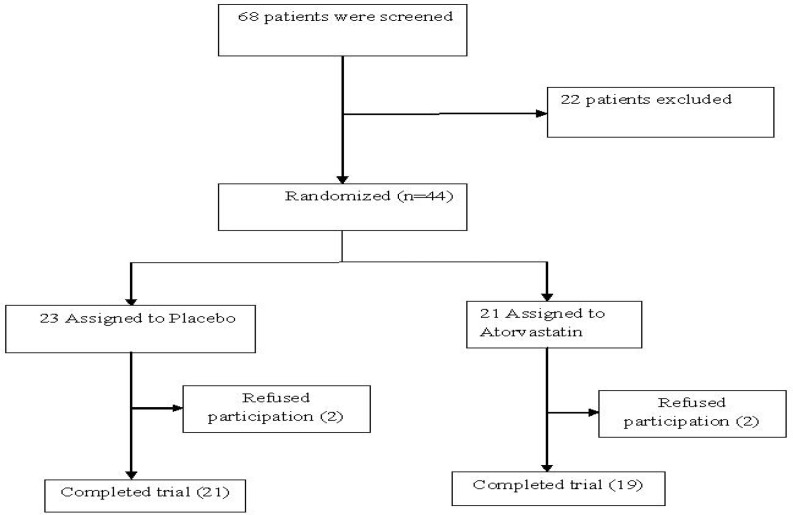
A chart of all patients screened for the study.

Forty patients completed the 6-week trial while 4 patients discontinued the trial. The treatment attrition did not differ between the two groups. Two patients withdrew from the trial in each study group (Figure 1).

*Effect on the SANS scores*

**Figure 2:**
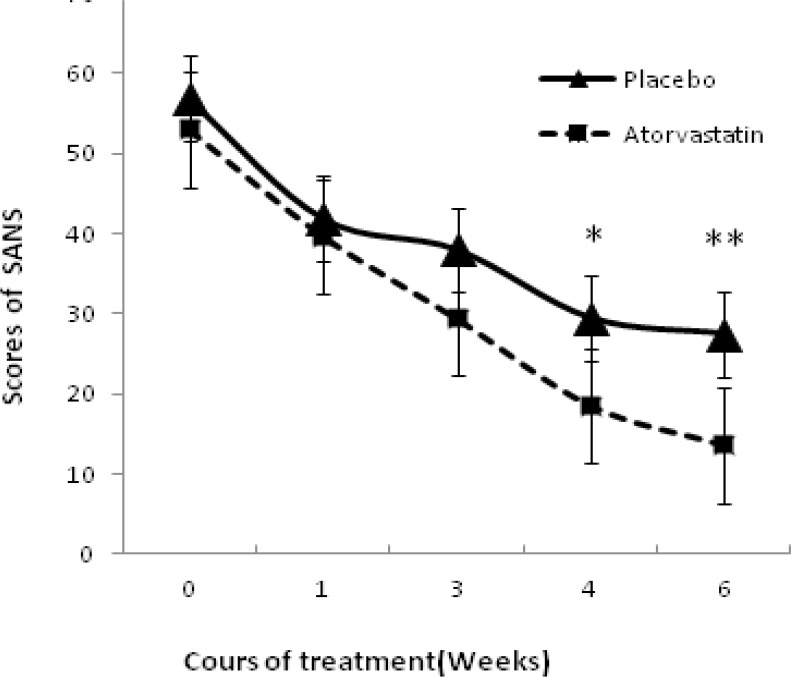
Mean ± SEM scores of two groups of patients on the SANS.

Repeated Measures ANOVA revealed a significant effect of time (F = 68.38, d. f = 1. P = 0.00).the effect of treatment was not significant (F = 0.001, dF = 1, P = 0. 974 > 0.1). Likewise, time-by-treatment interaction was not significant (F = 0.64, d. F. = 1, P = 0. 44 > 0.1) marginally no significant (t = 1. 85, P = 0.07). Mean scores of SANS decreased during treatment but there was no significant different between two groups (Figure 2). Obviously in the fourth and sixth weeks, differences were marginally significant (P = 0. 074 and P = 0. 068).

*P = 0. 074, **P = 0. 068 (Non Significant).

*Side Effect*

No patient discontinued treatment for adverse effects. The difference between two groups was not significant (Table 2).

**Table 2:** Patients with side effect

**Side effect**	**Placebo group (21)**	**Atorvastatin group (19)**	**P**
Weakness	1	1	Ns
Memory problems	1	2	Ns
Weight gain	0	1	Ns
Dizziness	1	2	Ns
Restlessness	2	1	Ns
Constipation	1	1	Ns
Daytime drowsiness	3	2	Ns

 Augmentation is a common strategy in psychiatry illness (17-19). Schizophrenia is a chronic disease that imposes considerable costs to the health system of any society (14). In addition, negative symptoms are one of the most important reasons for poor performance and the financial burden on society in the patients with schizophrenia and the reduction of these symptoms is a tough job for clinicians; therefore, to find a solution for this would be very helpful to these patients.

In particular, most of the complications are caused by negative symptoms and reported in patients. Signs and symptoms of schizophrenia indicate that the etiology of this disease is in the central nervous system and the volatility and the maladjustments in neurotransmitter cause these symptoms (20). One of the theories put forward to negative symptoms is the theory of inflammation in the CNS and other is neurotransmitters such as glutamate and NMDA and NO that can help in reducing negative symptoms by impacting on these factors.

NO is one of these important transmitters and regulators of physiological processes in the central nervous system that plays a role in the etiology of this disease by impacting on NMDA system (15-16) (6). Furthermore, inflammations in the central nervous system and the impacts on interleukin have been also mentioned as the causes of the disease (19). In this study, the effect of Atorvastatin with a dose of 20 mg was evaluated and compared to placebo, and ultimately a reduction of negative symptoms was observed during the course of six weeks. This reduction was more obvious especially at the end of fourth and sixth weeks while this decrease was not significant but in the symptoms was noticeable. Therefore, this was a continuous reduction and it can be guessed that the difference would be probably significant if the study continued to eight or 12 weeks. In the beginning of treatment, patients were obviously matched and they were not different in terms of the age, marital status, and the duration of the risk as well as the initial negative symptoms. During the study, no certain side effects were observed and the extra pyramidal in the two groups did not also increase. Due to the influence of Atorvastatin on inflammation, it can be concluded that probably inflammation is one of the causes for negative symptoms; in addition, negative symptoms as well as the influence of Atorvastatin on NO can be reduced by controlling it. It is worth nothing that No is one of the important neurotransmitter in the creation of the negative symptoms. The combination of anti psychotic drugs with such drugs that affect inflammation such as celecoxib or NO; we hope that negative symptoms of patients with schizophrenia would reduced more. The results of this trial must be examined in the shadow of its limitations. One of the defects of this study can be its time limitation (six weeks) for stabilization of the impact of this drug on the negative symptoms then it is better if the research would be conducted in the longer time period (eight to 12 weeks). The low number of samples (40 patients) is another restriction of study; so, more samples should be considered in the next studies. Another criticism of our work was that we did not monitor the effect of the drug on positive signs; so, in future studies the researchers can consider and apply this effect.

Atorvastatin could reduce the negative symptoms of patients with schizophrenia; however, unlike our initial theory, this decrease was not significant but these cuts though brief also can cause a clear horizon in the application of drugs with anti-inflammatory effects in patients with schizophrenia.

## References

[1] Jablensky A, Sartorius N, Ernberg G (1991). Schizophrenia: manifestation, incidence and course in different cultures. A World Health Organization ten-country study. Psychol. Med..

[2] Anderson NC (2000). Schizophrenia: the fundamental questions. Brain Res. Rev..

[3] Kirkpatrick B, Buchanan RW, Ross DE, Carpenter W (2001). A separate disease within the syndrome of schizophrenia. Arch. Gen. Psychiatry.

[4] Körschenhausen D, Hampel H, Ackenheil M, Penning R, Müller Fibrin N (1996). degradation products in postmortem brain tissue of schizophrenics: a possible marker for underlying inflammatory processes. Schizophr. Res..

[5] Müller N, Ackenheil M (1998). Psychoneuroimmunology and the cytokine action in the CNS: implications for psychiatric disorders. Prog. Neuro-Psychopharmacol. Biol. Psychiatry.

[6] Hans-Gert B, Bernhard B, Gerburg K (2005). The many faces of nitric oxide in schizophrenia. A review. Schizophr. Res..

[7] Bun-Hee L, Yong-Ku K (2008). Reduced plasma nitric oxide metabolites before and after antipsychotic treatment in patients with schizophrenia compared to controls. Schizophr. Res..

[8] Nawrocki JW, Weiss SR, Davidson MH, Sprecher DL, Schwarts SL, Lupien PJ, Jones PH, Haber HE, Black DM (1995). Reduction of LDL cholesterol by 25% to 60% in patients with primary hypercholesterolemia by Atorvastatin, a new HMG-CoA reductase inhibitor. Arterioscler. Thromb. Vasc. Biol..

[9] Vaughan CJ, Delanty N (1999). Neuroprotective properties of statins in cerebral ischemia and stroke. Stroke.

[10] Marz W, Koenig W (2003). HMG-CoA reductase inhibition: anti-inflammatory effects beyond lipid lowering?. J. Cardiovasc. Risk..

[11] American Psychiatric Association (2000). Diagnostic and Statistical Manual of Mental Disorders: DSM-IV-TR.

[12] Kay SR, Fiszbein A, Opler LA (1987). The positive and negative syndrome scale (PANSS) for schizophrenia. Schizophr. Bull..

[13] Andreasen NC (1982). Negative symptoms in schizophrenia. Definition and reliability. Arch. Gen. Psychiatry.

[14] King D, Knapp M, Patel A, Amaddeo F, Tansella M, Schene A, Koeter M, Angermeyer M, Becker T (2013). Angermeyer M and Becker T. The impact of non-adherence to medication in patients with schizophrenia on health, social care and societal costs. Analysis of the QUATRO study. Epidemiol. Psychiatric Sci..

[15] Rand MG, Li CG (1995). Nitric Oxide as a neurotransmitter in peripheral nervous: nature of transmitter and mechanism of transmission. Annu. Rev. Physiol..

[16] Virarkar M, Alappata L, Bradforda PG, Awada A (2013). L-arginine and nitric oxide in CNS function and neurodegenerative diseases. Critical Reviews in Food Science and nutrition.

[17] Mehdi sayyah M, Siahpoosh A, Khalili H, Malayeri A, Samaee H (2012). A Double-Blind, Placebo-Controlled Study of the Aqueous Extract of Echium amoenum for Patients with General Anxiety Disorder. Iran. J. Pharm. Res..

[18] Pakseresht S, Boostani H, Sayyah M (2011). Extract of valerian root (Valeriana officinalis L.) vs. placebo in treatment of obsessive-compulsive disorder: a randomized double-blind study. Journal of Complementary and Integrative Medicine.

[19] Ahmadi F, Assadi N (2013). Performance Evaluation of a Novel Potentiometric Membrane Sensor for Determination of Atorvastatin in Pharmaceutical Preparations. Iran. J. Pharm. Res..

[20] Keyhanfar F, Khani S, Bohlooli S (2014). Evaluation of Lipid-based Drug Delivery System (Phytosolve) on Oral Bioavailability of Dibudipine. Iran. J. Pharm. Res..

